# Mapping the dynamics of idiographic network models to the network theory of psychopathology

**DOI:** 10.3758/s13428-026-03009-w

**Published:** 2026-04-21

**Authors:** Ria H. A. Hoekstra, Jill de Ron, Sacha Epskamp, Donald J. Robinaugh, Denny Borsboom

**Affiliations:** 1 Department of Psychology, University of Amsterdam, Nieuwe Achtergracht 129B, 1018 WS Amsterdam, The Netherlands; 2 Department of Psychology, National University of Singapore, Singapore, Singapore; 3 Department of Psychiatry, Harvard Medical School, Massachusetts General Hospital, Boston, MA USA; 4 Department of Applied Psychology, Northeastern University, Boston, MA USA

**Keywords:** Stability landscape, Network connectivity, Idiographic network models, Network theory of psychopathology, Variability in symptom activation, Symptom severity

## Abstract

The network theory of psychopathology posits that mental disorders are stable states of symptom activation arising from causally interconnected symptoms, where individuals with more strongly connected symptom networks are at a higher risk of developing a mental disorder. Researchers have turned to idiographic network estimation to assess this theoretical position, yet it remains unclear whether the dynamics of these models align with the network theory. In this paper, we use stability landscapes to systematically map the parameters of idiographic network models onto the network theory. Specifically, we examine how the dynamics implied by the Ising model (with $\{0, 1\}$ and $\{-1, 1\}$ encodings) and the Graphical Vector Autoregressive (GVAR) model relate to symptom severity and variability. Our results show that only for a subset of parameter values in the $\{0, 1\}$ Ising model, higher connectivity is directly related to higher symptom severity, thereby aligning with the network theory. The $\{-1, 1\}$ Ising model only partially aligns with the network theory, as increased connectivity is related to both high and low symptom severity. In contrast, connectivity in the GVAR model is independent of symptom severity but instead reflects symptom variability over time. These findings demonstrate that the theoretical implications of network connectivity depend strongly on the chosen statistical model and its parameterization. Together, our results emphasize the need for systematic investigations that link theory to statistical models, and present stability landscapes as a useful tool to do so.

## Introduction

The way we conceptualize mental disorders and the statistical models we use to study them are intimately connected. For example, the introduction of the network theory of psychopathology (Borsboom, 2017) has gone hand in hand with the increasing popularity of statistical network models in psychology (e.g., Ising model, GGM, GVAR model; Robinaugh et al., 2020). However, theories and statistical models should not be conflated (Fried, 2020): One could estimate a latent variable model without assuming an underlying common cause, just as one could estimate a network model without assuming underlying causal interactions between variables (Borsboom et al., 2022; van Bork et al., 2021). Because statistical models are not necessarily coupled to psychological theories, it is important to consider how our theories map onto the dynamics of our statistical models (Haslbeck et al., 2022; Van Dongen et al., 2024). In this paper, we aim to relate the dynamics of statistical idiographic network models (i.e., person-specific network models that capture within-person dynamics, rather than between-person differences) to a fundamental principle of the network theory of psychopathology, namely that an individual’s symptom network reflects their propensity to develop a mental disorder.

The network theory of psychopathology characterizes mental disorders as a causal network of symptoms, where the presence of a mental disorder is defined as a stable state of symptom activation (Borsboom, 2017; Borsboom & Cramer, 2013). External shocks, or perturbations caused by factors outside the symptom network, such as losing one’s partner, can activate symptoms in the network, such as a depressed mood. Two characteristics of the symptom network determine how the system responds to these external perturbations. First, the *thresholds* of the symptoms determine the tendency of a symptom to be activated. Low (high) thresholds correspond to more (less) likely symptom activation.^1^ According to the network theory (Borsboom et al., 2017), thresholds for most of the symptoms should be high, since on a population level most symptoms are uncommon and symptom severity distributions are typically skewed to the right (Sturt, 1981). Second, the *connectivity* of the network (i.e., the presence and the strength of the relations between symptoms) determines how easily a symptom activates other symptoms: Symptom activation in a strongly and fully connected network triggers the activation of many other symptoms, resulting in cascading feedback effects that maintain symptom activation over time. Conversely, symptom activation within a sparsely connected network does not necessarily exhibit this effect; the likelihood of a single symptom triggering a cascade of activity in many other symptoms is small due to the relatively weak causal relations (Cramer et al., 2016).

Researchers increasingly employ intensive longitudinal data techniques, such as experience sampling methods, to determine an individual’s network characteristics. Various idiographic statistical methods can be used to examine this data, of which the graphical vector autoregressive (GVAR) model is currently the most popular (Bringmann, 2021; Burger et al., 2022). Based on the network theory of psychopathology, many researchers have examined whether individuals who have or are at risk of developing a mental disorder have higher idiographic network connectivity than those who do not. However, the empirical literature examining the relationship between connectivity and psychopathology has shown mixed results. While many network studies reported that increased network connectivity is associated with *increased* levels of psychopathology (e.g., Lydon-Staley et al., 2019; McElroy et al., 2019; Pe et al., 2015; Schweren et al., 2018; Shin et al., 2022; van Borkulo et al., 2015; Wigman et al., 2015), other work finds no association between network connectivity and psychopathology (e.g., Frumkin et al.,^2021^; Kelley et al., 2023; Lunansky et al., 2023). Notably, some studies report different conclusions depending on the level of analysis. For instance, De Vos et al. (2017) found an association at the group-average (fixed-effect) temporal network level, but no association between network density and psychopathology when examining individual-specific (random-effect) networks.

**Fig. 1:**
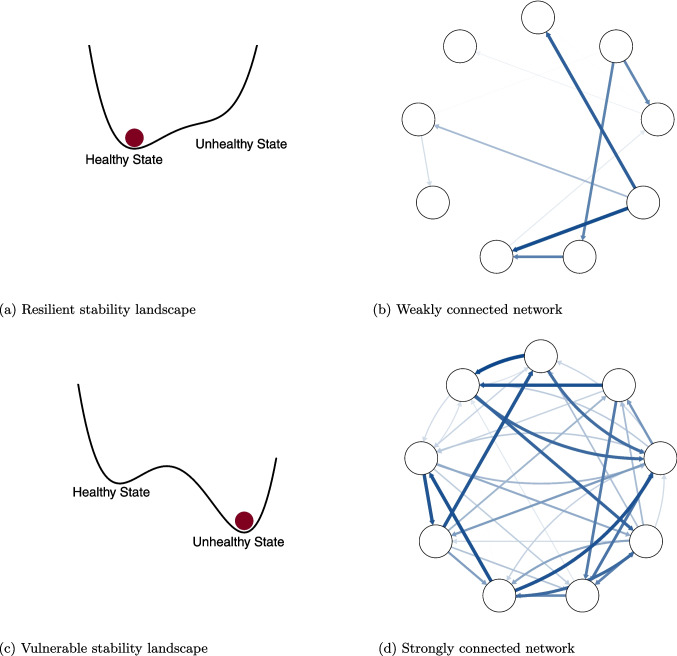
The network theory of psychopathology posits that individuals vulnerable to developing a mental disorder have a more strongly connected symptom network than resilient individuals, and therefore their stability landscapes differ. (**a**) A resilient stability landscape against developing a mental disorder. (**b**) The corresponding symptom network has low connectivity. If an external shock activates one of the symptoms (i.e., the ball is perturbed to the right side away from the stable state), due to the weak connections in the network other symptoms will not remain activated in response, and the system will recover rapidly to its original state. (**c**) A stability landscape that is vulnerable to ending up in the unhealthy phase. (**d**) The corresponding symptom network has high connectivity. In a densely connected network, the activation of one symptom easily cascades to the activation of other symptoms, and is more likely to cause the system to move its state over the so-called tipping point into the unhealthy phase

These mixed results illustrate that translating the network theory of psychopathology into statistical networks is not straightforward. Whether a statistical network model aligns with network theory depends on whether the statistical network can represent dynamics in a way consistent with the theory. Therefore, researchers must have tools to examine the connection between the network theory and idiographic network models so that they can assess what they *would* expect to see in the statistical model *if* the connectivity or thresholds of the model change. To that end, stability landscapes are highly useful to represent model behavior (Cui et al., 2025), because they offer an easy-to-understand visual tool to study the long-term dynamics of a model based on all parameters simultaneously: In a single visualization, a stability landscape gives insight into the effect of external shocks, thresholds, and connectivity.

In this paper, building upon the work by Cui et al. (2025, 2023a, 2023b, 2023c), we use and develop stability landscapes to examine the connection between idiographic network models and the dynamics implied by the network theory of psychopathology. The paper is structured as follows. First, we introduce stability landscapes as a tool to gain insight into the dynamics of a system (here: A symptom network). Second, we conduct a simulation study where we systematically vary network characteristics (i.e., thresholds or means and connectivity) and use stability landscapes to investigate the impact of these characteristics on the dynamics displayed by two idiographic network models, namely the Ising model (both within the $\{0,1\}$ domain and the $\{-1,1\}$ domain) and the GVAR model. We show that the dynamics of the Ising model within the $\{0,1\}$ domain can be translated to the network theory, but only for specific parameter values. Both the dynamics of the Ising model within the $\{-1,1\}$ domain and the GVAR model cannot be directly translated to the network theory.

## Stability landscapes

Stability landscapes summarize the dynamics of a system by visualizing the tendency of the system to be in each possible state. For instance, when we define the system to be a symptom network, a possible state might correspond to all symptoms being present, while another state might correspond to no symptoms being present. In psychology, stability landscapes have mainly been used as a conceptual illustration, the so-called *ball-in-cup* metaphor (Bringmann et al., 2023; Heino et al., 2023; Kalisch et al., 2019; Olthof et al., 2023; Wichers et al., 2019). Figure 1 Panel (a) and (c) show two conceptual stability landscapes: The valleys represent states in which the system is most likely to end up. The deeper the valley, the more stable the state. The red ball represents the system’s current state: The ball can roll over the landscape due to perturbations that represent the influence of external shocks (e.g., adverse life events). How these perturbations change the current state of the system depends on the shape of the landscape. When the ball is in a stable state (valley), a perturbation temporarily disturbs the system, but the ball moves back to the original stable state over time. The more stable a state (the deeper the valley), the more difficult to transition to another state (e.g., only a significant perturbation can push the ball up the hill to the other valley). The point of transition from one stable state to the other, as represented by a hill, is called a tipping point as a small perturbation pushes the state of the system (i.e., the ball) towards an alternative stable state.

### Linking the network theory to stability landscapes

In the network theory of psychopathology, a psychological disorder corresponds to a system that is in a stable, self-sustaining state of symptom activation, while recovery corresponds to a transition to a healthier, stable state of no or little symptom activation. Accordingly, stability landscapes have often been used as a metaphorical illustration of the two main predictions of the network theory. First, the network theory predicts that in networks with high thresholds, increasing network connectivity leads to a self-maintaining stable state represented by stability landscapes where the unhealthy state has a deeper and steeper basin (Borsboom et al., 2017). Clinically, increased stability of the unhealthy state represents more persistent symptom activation.

Second, the network theory predicts that in networks with high thresholds, a more strongly connected network leads to stability landscapes where the tipping point (hill) between the stable states becomes more prominent, and the behavior of the system becomes increasingly discontinuous. Greater discontinuity in the system implies that transitions between a healthy and an unhealthy state become more abrupt and harder to reverse. Clinically, increased discontinuity represents more resistance to treatment efforts such as therapy or medication. That is, for a person with a low connected symptom network, the system responds proportionally to external shocks: The ball can roll to an unhealthy state but once the external shock is removed, the system returns back to its original, healthy state. However, for a person with a highly connected symptom network, the system displays non-linearity and responds disproportionately to external shocks. Such that, if the person is near a tipping point, small perturbations can have large effects. Once the tipping point between the two stable states is reached, the system rapidly moves to the unhealthy state. Since the unhealthy state itself is stable, the disappearance of the external shocks will not cause the system to return to its original healthy state.^2^

In summary, a network with high thresholds and low connectivity, see Panel (b) of Fig. 1, gives rise to a stability landscape that is characterized by a single stable state, corresponding to a healthy state of low symptom activation, see Panel (a) of Fig. 1. In contrast, a network with high connectivity, see Panel (d) of Fig. 1, gives rise to a stability landscape that is characterized by two alternative stable states, a healthy and an unhealthy state, and features a tipping point, see Panel (c) of Fig. 1. For such individuals, the healthy state is a shallow basin and the unhealthy state is characterized by a deep and steep basin; *ipso facto* states of persistent symptom activation, i.e., mental disorders, are both accessible and stable.

## Methods

Here we map the two predictions of the network theory to idiographic network model dynamics by constructing stability landscapes (Cui et al., 2023a, b, c). We focus on two idiographic network models that can be estimated for an individual on longitudinal, multivariate data: The Ising model for binary data and the GVAR model for continuous data. Specifically, we present a simulation study where we vary both the connectivity and the thresholds for the Ising model or the means for the GVAR model to systematically investigate the effect of these different parameterizations on symptom *severity* and *variability* in symptom activation. This allows us to determine the extent to which the predictions of the network theory align with the dynamics observed in statistical methods used to estimate such idiographic networks.

### Ising model

The Ising model defines the probability of a node $x_i$ to be “on" (encoded as 1) or “off" (encoded as 0 or $-1$) given all other nodes in the network. In psychological network models, nodes correspond to symptoms, and a node being “on" or “off" corresponds to a symptom being present or absent, respectively. The probability for each node to be “on" is based on its threshold ($\tau$) and the strength of the connections between neighboring nodes, which is represented by edge weights ($\omega$). This implies the following probability over a certain configuration of nodes $\textbf{x}$^3^:

1 $$\begin{aligned} P(\boldsymbol{X} = \boldsymbol{x}) = \frac{\exp \left( \displaystyle \sum _{i} \tau _{i} x_i + \displaystyle \sum _{<i,j>}{\omega _{i,j} x_j x_i}\right) }{\displaystyle \sum _{\boldsymbol{X}} {\exp \left( \displaystyle \sum _{i}{\tau _{i} x_i} + \displaystyle \sum _{<i,j>}{\omega _{i,j} x_j x_i}\right) }}, \end{aligned}$$

where $\textbf{X}$ is a vector representing all possible states of the Ising model, $x_i$ represents the state of node *i*, and $\tau _{i}$ is the threshold of node *i*, which represents the tendency of node *i* to be activated independently of the other nodes in the network. A threshold of zero is neutral; a negative threshold corresponds to the tendency for a symptom to be off, and a positive threshold corresponds to the tendency for a symptom to be on. Note that the thresholds in the Ising model are mirrored relative to the thresholds used in the original formulation of the network theory of psychopathology, in which a low threshold corresponds to the tendency for a symptom to be on (Borsboom et al., 2017).

The edge weights are represented by matrix of size $n \times n$ nodes, where each edge weight $\omega _{i,j}$ represents the strength of alignment between two nodes (Ising, 1925). However, the exact interpretation of the edge weights is dependent upon the encoding of the nodes (Haslbeck et al., 2021b): when nodes are coded as $\{0, 1\}$ the strength of the positive edge weight indicates the increased probability of two nodes to be in state $\{1, 1\}$ relative to the probability of all other states $\{0, 1\}$, $\{1, 0\}$, and $\{0, 0\}$. In contrast, when nodes are encoded as $\{-1, 1\}$, an edge weight above zero indicates that nodes align with each other in either direction, either $\{1, 1\}$ or $\{-1, -1\}$, and an edge weight below zero indicates that opposite labels align with each other, $\{-1, 1\}$. As the dynamics of the Ising model are dependent upon the encoding of the nodes, we investigate the dynamics of both the Ising model in the $\{0,1\}$ and the $\{-1,1\}$ domain.^4^

### GVAR model

The GVAR model defines the value of each variable at time *t* as a linear combination of itself (so-called auto-regressive or lagged effects) and all other variables in the model (so-called cross-lagged effects) on the previous time point $t-1$ as follows (Epskamp et al., 2018b; Hamilton, 1994):

2 $$\begin{aligned} \pmb {y}_{t}&= \pmb {\mu } + \pmb {B}(\pmb {y}_{t-1}-\pmb {\mu }) + \pmb {\varepsilon }_{t} \end{aligned}$$

3 $$\begin{aligned} \pmb {\varepsilon }_{t}&\sim N \left( 0, \pmb {\Sigma }\right) \end{aligned}$$

where $\pmb {y}_{t}$ denotes a vector of responses of a given person at time *t*, $\pmb {\mu }$ is the mean of the distribution of each variable, $\pmb {B}$ is a matrix containing the lagged and cross-lagged relations also known as the temporal network, $\pmb {y}_{t-1}$ is the vector of responses of person at a previous time point $t-1$ and $\pmb {\varepsilon }_{t}$ is a Gaussian noise process with a time-invariant positive definite covariance matrix $\pmb {\Sigma }$ which can be used to encode a contemporaneous network (Epskamp et al., 2018a, b).^5^ This contemporaneous network represents the relationship between the variables within the same measurement window that cannot be accounted for by temporal effects.

In contrast to the Ising model, the GVAR model does not contain a parameter that directly translates to the thresholds as formulated in the network theory of psychopathology. Thresholds determine the likelihood of symptom activation in a binary system. The GVAR model, by contrast, describes continuous fluctuations around a mean level and, therefore, does not incorporate threshold parameters. Instead, the GVAR model features a mean parameter $\mu _{i}$ that controls the location of the distribution of variable *i*. If one were to formulate a network theory of psychopathology using continuous symptoms, this parameter could play an analogous role to the threshold in the Ising model, as the higher the mean for a given symptom, the higher its intensity, and thus the more the symptom is “activated”. See Table 1 for an overview of the different interpretations of the network characteristics in the network theory, the Ising model, and the temporal network of the GVAR model.

**Table 1:** Connecting network characteristics to statistical models

Network theory	Ising model	GVAR model
thresholds	$\tau$	$\mu$
low = *higher* symptom activation	positive = *higher* symptom activation	high = *higher* symptom activation
high = *lower* symptom activation	negative = *lower* symptom activation	low = *lower* symptom activation
connectivity	$\frac{2}{n(n-1)} \sum _{i=1}^{n} \sum _{j=i+1}^{n} | \omega _{ij} |$	$\frac{1}{n^2} \sum _{i=1}^{n} \sum _{j=1}^{n} | \beta _{ij} |$

Thresholds in the network theory of psychopathology refer to the amount of external force a symptom needs in order to become active (switch from being absent to being present). According to the network theory, a high threshold relates to low symptom activation and vice versa. Thresholds in the Ising model can be interpreted as the probability of symptom activation. Meaning symptoms with a positive threshold have a higher probability of being active. For the GVAR model, the mean of the symptom refers to symptom activation, where a higher mean is indicative of high symptom activation. Connectivity is defined as the weighted average strength of the estimated network model for both the Ising model and the GVAR model

### Simulation setup

To examine how statistical idiographic network models correspond to the theory-implied network dynamics of psychopathology and assess the extent to which the network theory’s predictions match the dynamics observed in statistical methods used to estimate idiographic networks, we use a simulation approach.^6^ In the simulation study reported here, we vary the $\tau$ parameters for the Ising model and the $\mu$ for the GVAR model and the network connectivity and we investigate their effect on the stability landscape.

In order to achieve this goal, our simulation study follows three steps. In the first step, we construct a baseline network for the Ising model in the $\{0,1\}$ and $\{-1,1\}$ domain and the GVAR model. Next, we vary either the $\tau$ parameters or the $\mu$ parameters and the connectivity of these baseline networks. In the second step, we construct a stability landscape for each of the different model parameterizations. Stability landscapes visualize a potential energy function *U* (Cui et al., 2023a, b, c). The potential energy represents the probability of the system to be in a certain state (here: the number of active nodes in the Ising model and the sum score of the variables in the GVAR model), with lower energy representing a higher probability for the system to be in that state. In the Appendix A we specify how we get to the stability landscapes. In the third step, we inspect the stability landscapes to see whether increasing network connectivity indeed leads to landscapes where (1) the healthy state becomes less stable, and the unhealthy state becomes more stable, and (2) the tipping point becomes more severe. The code to generate the analyses is available on the Open Science Framework (OSF) repository at https://osf.io/rgzqv/.

#### Constructing the baseline networks

To construct the baseline Ising networks, we use data from the Virginia Adult Twin Study of Psychiatric and Substance Use Disorders (VATSPSUD; Kendler & Prescott, 1999). This data set includes binary variables for nine depressive symptoms of 8, 973 twins from the Mid-Atlantic Twin Registry.^7^ To estimate the Ising model we used the function Ising from the *psychonetrics* package. In the original data set, the variables are encoded as 1 (symptom present) and 0 (symptom absent). When estimating the baseline Ising network within the $\{-1,1\}$ domain, we recode absent symptoms as $-1$.

In line with the Ising network, we also construct a baseline temporal GVAR network ($\pmb {B}$) with nine variables. We sample the weights for the cross-lagged relations from a uniform distribution between 0.1 and 0.2, and the autoregressive relations from a uniform distribution between 0.2 and 0.4, so that the temporal network has stronger autoregressive effects than cross-lagged effects. In line with the network theory, all connections between nodes in the network are positive. We construct the contemporaneous network using the genGGM() function from the *bootnet* package (Epskamp & Fried, 2023) with only positive edges.

For both types of network models, we multiply the baseline network by a constant, which we refer to as the *multiplier*. The multiplier determines the network’s connectivity; for the Ising model, we multiply the baseline network by 0.9, 1, or 2. For the GVAR model, we multiply the temporal network either by 0.5, 1, or 1.2, while the contemporaneous network remains the same in all three conditions. In addition, we adjust the threshold parameter ($\tau$) for the Ising model and the means ($\mu$) for the GVAR model. For the Ising model, we study three different threshold conditions, namely baseline thresholds (as these are negative, they represent the tendency of all symptoms to be off), the baseline thresholds multiplied by 2 (leading to even more negative thresholds), and positive counterpart of the baseline thresholds (by multiplying the thresholds by $-1$; representing the tendency of all symptoms to be on). Note that the condition with positive thresholds in the Ising model is inconsistent with network theory, which predicts that symptom thresholds tend to be negative. For the GVAR model, we study three different mean conditions, namely $\mu = 0$, $\mu = 3$ and $\mu = 6$.

**Fig. 2:**
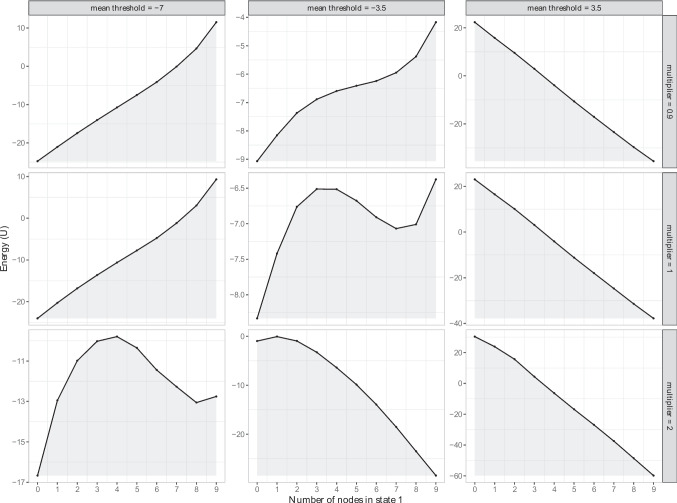
The potential stability landscapes for the Ising model, where absent symptoms are coded as 0 and present symptoms as 1. We varied the network density and the thresholds. The *x*-axis represents the state of the system, namely the number of active symptoms, and the *y*-axis the potential energy (U) of that state. The lower the potential energy, the more stable the state is

For our simulation, we end up with three different network models: the Ising model in the $\{-1,1\}$ domain, the Ising model in the $\{0,1\}$ domain, and the GVAR model. Thus, in total, we construct 27 stability landscapes: 3 (type of network models) $\times$ 3 (thresholds or $\mu$’s) $\times$ 3 (connectivity multipliers).

## Results

### Ising model dynamics

#### The {0,1} domain

Figure 2 shows nine stability landscapes with varying parameter values of the Ising model within the $\{0,1\}$ domain. The *x*-axis shows the number of active symptoms and the *y*-axis shows the value of the energy function, *U*, where low energy indicates a higher probability for the system to be in that state. The columns indicate the mean of the threshold parameters (i.e., mean $\tau \in \{-7, -3.5, 3.5\}$), while the rows indicate the multiplier that was used to either make the baseline network sparser (i.e., $multiplier = 0.9$) or denser (i.e., $multiplier = 2$).

The results show that when the thresholds are positive (mean $\tau$ = 3.5, see right column of Fig. 2), the system has one stable, unhealthy state (i.e., all symptoms are active). For the threshold conditions that are in line with the network theory of psychopathology, namely those with negative thresholds (left and middle column of Fig. 2), there are three consequences of increasing connectivity. First, increasing the network connectivity results in a deeper, unhealthy stable state and a more shallow healthy stable state. Second, as the connectivity increases, the unhealthy stable state becomes more severe (i.e., a higher number of nodes are in state one). Third, as connectivity increases, the number of stable states changes: there is one stable state when network connectivity is low (multiplier = 0.9), but two stable states appear at higher connectivity. Although in the condition with the highest network connectivity and with mean thresholds of -3.5, the healthy stable state almost disappears. Thus, initially, as connectivity increases, a pattern of increasing discontinuity emerges. That is, at lower levels of connectivity, the Ising model may have only one stable state and exhibit a relatively smooth transition between different levels of symptom activation. However, as connectivity increases, a tipping point (as indicated by a hill in the landscape) emerges. As connectivity increases further, only one unhealthy stable state remains.

In summary, the results within the $\{0,1\}$ domain, at least within the range of plausible parameters, are in line with the two predictions of the network theory, see Table 2. Furthermore, there is an interaction effect between thresholds and connectivity: the more negative the thresholds, the more connectivity is needed in order to obtain two instead of one stable state(s). Thus, networks with the same connectivity can behave differently if the thresholds are different.

#### The {-1,1} domain

**Fig. 3:**
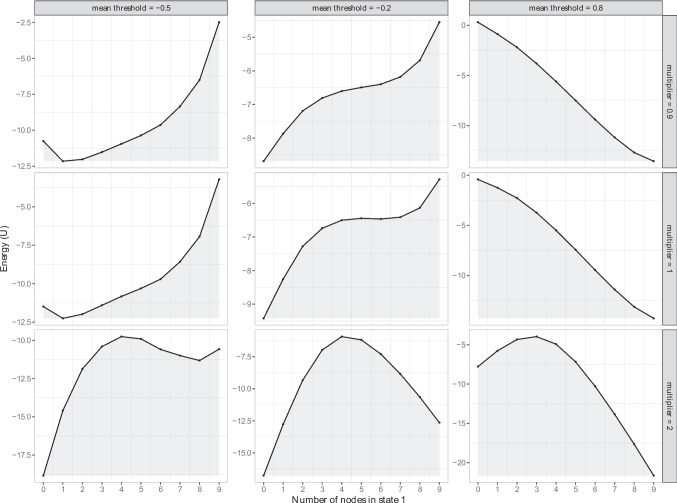
The potential stability landscapes for the Ising model, where absent symptoms are coded as $-1$ and present symptoms as 1. We varied the network’s connectivity and the thresholds. The *x*-axis represents the state of the system, namely the sum score of the variables, and the *y*-axis the potential (U) of that state. The lower the potential, the more stable the state is

Figure 3 shows nine stability landscapes with varying parameter values of the Ising model within the {-1,1} domain. The *x*-axis shows the number of active symptoms and the *y*-axis shows the value of the energy function, *U*. The columns indicate the mean thresholds (i.e., mean $\tau \in \{-0.5, -0.2, 0.8\}$), while the rows indicate the multiplier that was used to either make the baseline network sparser (i.e., $multiplier = 0.9$), or denser (i.e., $multiplier = 2$).

The stability landscapes have either one or two stable states, depending on the mean thresholds and the connectivity. The threshold condition with a mean $\tau = -0.5$, see left column in Fig. 3, shows that when connectivity is low (multiplier = 0.9) or at baseline (multiplier = 1), the healthy state (i.e., the number of active symptoms is one) is the most likely to occur. However, with increased connectivity (multiplier = 2) a new, unhealthy stable state emerges (i.e., the number of active symptoms is eight).

**Fig. 4:**
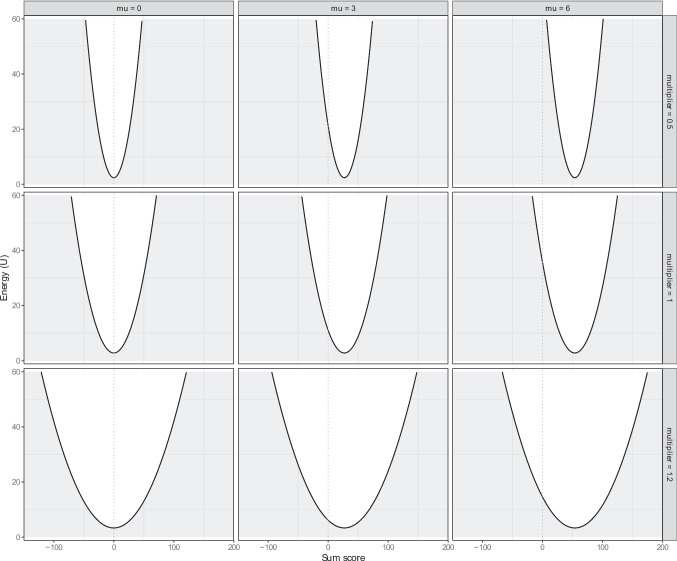
The potential stability landscapes of the GVAR model, where we varied the density of the temporal network $\beta$ and the $\mu 's$. The *x*-axis represents the state of the system, namely the sum score of the variables, and the *y*-axis the potential (U) of that state. The lower the potential, the more stable the state is

When the mean thresholds are -0.2 and network connectivity is high (multiplier = 2), the Ising model shows two stable states: One basin appears around zero active symptoms and one basin appears around nine active symptoms, see the plot at the bottom of the middle column in Fig. 3. Given mean thresholds of -0.2, with increasing connectivity *both* stable states become more resilient to perturbations. Thus, similar to the Ising model within the $\{0,1\}$ domain, the unhealthy state becomes more stable with increasing connectivity; however, in contrast to the Ising model within the $\{0,1\}$ domain, the healthy stable state also becomes more stable. That is, due to the encoding increasing connectivity not only renders two nodes in a $\{1,1\}$ state more likely but also in the $\{-1,-1\}$ state (Haslbeck et al., 2021b). When the mean thresholds are positive ($\tau = 0.8$), increasing connectivity leads to the appearance of a second (healthy) state. In this condition, stronger connectivity leads to a system that is more likely to be in a healthy state than a less connected network.

### GVAR model dynamics

Figure 4 shows nine stability landscapes with varying parameter values of the GVAR model. The columns indicate the difference in mean parameters (i.e., $\mu \in \{0, 3, 6\}$), while the rows indicate the difference in the multiplier that was used to either make the baseline network more sparse (i.e., $multiplier = 0.5$), or more dense (i.e., $multiplier = 1.2$).

Because the GVAR model is linear, the stability landscape has only one stable state. The mean, $\mu$, determines the position of the stable state. When $\mu = 0$, we find a stable state at the sum score of 0. When we increase the mean, the position of the stable state shifts to the right, regardless of the network connectivity. The network connectivity determines the steepness of the valley in the landscape, i.e., the amount of variation possible around the stable state. When connectivity is low ($multiplier = 0.5$), there is less variation around the stable state as indicated by a steep valley. When connectivity is high ($multiplier = 1.2$), there is more variation around the stable state, as indicated by a more shallow stable state.

### Discussion simulation results

Table 2 provides an overview of the simulation results. Regarding the dynamics of the Ising model, we see that the qualitative behavior of the model depends on the encoding of the variables: We found that for the conditions we investigated, only a subset of the parameter space of the Ising model within the $\{0,1\}$ domain corresponds to the dynamics predicted by the network theory of psychopathology. The Ising model within the $\{-1,1\}$ domain only partly aligns with the network theory: Increasing network connectivity leads to increasingly discontinuous behavior, but both the healthy and the unhealthy state become more stable. As the GVAR model is a linear model, it only shows one stable state and thus both predictions of the network theory cannot be directly translated to the GVAR model.

**Table 2:** Recovery of the dynamics for the Ising model and the GVAR model as expected by the network theory of psychopathology

Predictions of the network theory of psychopathology	Ising model $\{0,1\}$ domain	Ising model $\{-1,1\}$ domain	GVAR model
When connectivity is increased, the healthy state becomes less stable, and the unhealthy state becomes more severe and stable	$\checkmark$	$\times$	$\times$
When connectivity is increased, the system becomes more discontinuous	±	$\checkmark$	$\times$

## Discussion

Ever since the introduction of the network theory of psychopathology, methodological advances to estimate idiographic network models have developed in parallel (Borsboom, 2017; Robinaugh et al., 2020). However, whether a *statistical* method for identifying symptom networks in an empirical study aligns with a *theory* of how networks evolve depends on whether the statistical network is capable of representing dynamics in a way that is consistent with the theory. In this paper, we systematically investigated the link between the network theory of psychopathology and the parameterization of different statistical idiographic network models using stability landscapes. In particular, we constructed stability landscapes to map the dynamics of the Ising model and the GVAR model to the two predictions of the network theory of psychopathology: More strongly connected idiographic networks lead to stability landscapes where (1) the healthy state becomes less stable, and the unhealthy state becomes more stable and severe, and (2) the behavior of the system becomes increasingly discontinuous. Our results indicate that translating the network theory of psychopathology into statistically estimated idiographic network models depends heavily on the chosen model (e.g., an Ising model or a GVAR) and its parameterization.

Using stability landscapes, we have shown that only for a specific subset of parameters the Ising model within the $\{0,1\}$ domain aligns with the two predictions of the network theory of psychopathology. This indicates that the network theory’s characterization of highly connected networks as networks of individuals who are vulnerable to developing a mental disorder depends on fitting an Ising model in the $\{0,1\}$ domain. This finding is expected, as this model is equivalent to the one used by Cramer et al. (2016), which underlies the network theory of psychopathology.

The dynamics of the Ising model within the $\{-1,1\}$ domain only partially align with the network theory of psychopathology. In accordance with the network theory, increased network connectivity leads to increasingly discontinuous behavior. However, according to the network theory, increased connectivity should only make the unhealthy state more stable, while in the Ising model within the $\{-1,1\}$ domain, both the healthy (low symptom activation) and unhealthy (high symptom activation) states become more stable. Such dynamics seem more characteristic of attitudes, which can be characterized by polarization, rather than of mental disorders such as depression (Dalege et al., 2016; Haslbeck et al., 2021b).

The dynamics of the GVAR model do not align with the network theory of psychopathology. A fundamental assumption of the GVAR model is that variables fluctuate around a stable mean (Bringmann et al., 2024; Burger et al., 2022; Zhang et al., 2025). This inherently limits its capacity to represent multiple stable states, such as the dichotomy between healthy and unhealthy states, as depicted in the network theory of psychopathology. Consequently, while constructing stability landscapes of a GVAR model can provide insights into behavior within a stable state, it cannot display transitions between different stable states.

### Implications and practical considerations

In line with the empirical findings of Lunansky and Hoekstra (2023) and Kelley et al. (2023), our results indicate that higher connectivity in the GVAR model is linked to greater variability of the variables within the network. From a clinical perspective, our findings suggest that network connectivity should not be inherently interpreted as “good” or “bad", but should be understood in relation to the model’s underlying dynamics. For instance, a network showing high connectivity might be taken to indicate increased vulnerability, yet this interpretation can change entirely when thresholds for the Ising model or means for the GVAR model are taken into account. In practice, this implies that researchers should report all relevant parameters (e.g., thresholds for Ising models, and means or intercepts for GVAR models, in addition to the edge weights). At present, thresholds and means or intercepts are often omitted or not discussed, which, we have shown, can obscure the interpretation of network connectivity.

Importantly, these interpretational challenges do not arise only at the level of parameter reporting, but they can already emerge from mismatches between the network theory, the statistical model, and the study design. In addition to determining whether a statistical network model can represent dynamics consistent with network theory, it is equally important to consider whether the study design aligns with the theoretical claims. At a minimum, alignment between the network theory and statistical network models depends on (a) whether the assessed variables correspond to the symptoms that are hypothesized to play a role in the network theory, and (b) whether the level of the data (e.g., cross-sectional versus longitudinal) matches the level at which the theory is formulated.

When considering this alignment, two recurring inconsistencies in the empirical literature become apparent. First, nodes in network theory correspond to symptoms as encoded in diagnostic manuals such as the DSM-5 (Borsboom et al., 2017). In contrast, in many intensive longitudinal studies using network modeling, nodes correspond to variables that assess the fluctuation in momentary mood states rather than, or in addition, to symptoms (e.g., Kelley et al., 2023; Lunansky et al., 2023; McGhie & McNally, 2023; Pe et al., 2015; Piccirillo & Rodebaugh, 2022; Shin et al., 2022; Wigman et al., 2015).^8^ Second, while the network theory is explicitly formulated at the within-person level, empirical studies frequently rely on cross-sectional data or between-person associations. This mismatch in data level further complicates the interpretation of statistical network findings as evidence for, or against, the causal mechanisms proposed by the theory. These inconsistencies highlight that questions inspired by network theory cannot be answered independently of the level and nature of the available data.

The choice of a statistical method should ideally be guided by the type of question being asked. For instance, different idiographic network models capture different aspects of psychological dynamics: Stationary GVAR models may be better suited for studying short-term fluctuations within a stable state, whereas the Ising model, time-varying VAR, or Hidden Markov models are more appropriate for examining transitions between states (Aarts & Haslbeck, 2025; Finnemann et al., 2021b; Haslbeck et al., 2021a). More broadly, not every question inspired by the network theory of psychopathology requires a statistical network model to be estimated. Some questions may be better explored through theoretical or simulation-based work, or using different statistical techniques such as a *t*-test.

While we have shown that the dynamics of the GVAR model do not align with the network theory of psychopathology, such models can still provide valuable insights within clinical research. Specifically, GVAR models could help identify robust empirical patterns in data (Robinaugh et al., 2020; Ryan et al., 2023), such as *inertia*—the degree to which individuals’ emotions persist from one moment to the next (Hamaker & Grasman, 2015; Koval & Kuppens, 2012). In the GVAR model, inertia is reflected in the autoregressive parameters: values closer to zero indicate a faster recovery, indicating little carryover from one measurement occasion to the next. Conversely, values closer to one imply a slower recovery rate, indicating a considerable carryover effect from one measurement occasion to the next. High emotional inertia has been linked to several mental health factors, such as depression, neuroticism, and low self-esteem (Koval et al., 2012; Kuppens et al., 2010; Suls et al., 1998). In addition, constructed stability landscapes based on GVAR models can provide an intuitive visual representation of the magnitude of fluctuations around a person’s stable state and facilitate comparisons between individuals in terms of system variability.

### Limitations and future outlook

While our results underscore the significant impact of network model parameterization on the resulting dynamics, the primary limitation of our study is that we examined a limited range of network models and parameter values. Future work should therefore explore how different parameterizations affect model behavior more broadly. With the accompanying code presented online and the R-package isinglandr (available at https://cran.r-project.org/package=Isinglandr; Cui et al., 2023a), researchers can visualize stability landscapes of different parameterizations, such as for their own estimated Ising or GVAR models. Furthermore, researchers could develop methods to explore the stability landscapes of different types of idiographic network models, such as the time-varying VAR model (TV-VAR; Haslbeck et al., 2021a), threshold VAR model (T-VAR; Grynkiv & Stentoft, 2018), multilevel integrated VAR model (ML-VARI; Henry et al., 2022), mean switching hidden Markov model (HMM; Hamaker et al.,^2016^; Rabiner, 1989).

Another possible approach to investigate a range of parameterizations in the Ising model is by using the mean-field approximation, which shows how the location of the stable states shifts in response to changes in parameter values (Van der Maas, Dalege & Waldorp, 2020). However, the mean-field approximation relies on simplifying assumptions such as equal thresholds for each node and a densely connected network with positive connections (van der Maas, Waldorp & Borsboom, 2025). Although the assumption of positive connections in symptom networks holds in many empirical network studies (Robinaugh et al., 2020) and is in line with the network theory, assuming equal thresholds across symptoms seems less realistic. For example, the symptom ‘suicidal ideation’ is likely to have a higher threshold than the symptom ‘depressed mood’, as it is rarer and typically requires more severe distress to become activated. To avoid these assumptions, rather than relying on a mean-field approximation, we explored the effects of different model parameterizations on the Ising model using stability landscapes in the current paper.

A second limitation is that we simulate under a stationary GVAR model, assuming normally distributed variables. However, GVAR models are frequently applied to affect data, which often deviate from normality; for instance, negative affect variables tend to be highly skewed to the right (Haslbeck et al., 2023). Furthermore, both negative and positive affect variables are bounded at zero. That is, affect states can be absent but not negative. Such distributional constraints may influence the interpretation of network connectivity with regards to psychopathology. For example, increased connectivity in a network with highly skewed negative affect variables could indicate that negative affect was more often prevalent, possibly corresponding to increased levels of psychopathology. Future studies could therefore examine how such distributional characteristics influence the dynamics displayed by the GVAR model and their theoretical interpretation.

### Conclusion

Our results urge caution in translating concepts rooted in the network theory of psychopathology directly onto the dynamics of idiographic network models. To that end, stability landscapes can serve as a useful tool to bridge this gap, providing a framework to explore the connection between substantive theory and statistical network models.

## Data Availability

The dataset used in this study is not publicly available due to data sharing restrictions. However, all analysis code and materials necessary to reproduce the results are publicly available via the Open Science Framework (OSF): https://osf.io/rgzqv/.

## Appendix A Constructing stability landscapes

Stability landscapes visualize a potential energy function (Cui et al., 2023a, b, c). A potential energy function defines the probability of the system being in a certain configuration (e.g., the observed values of the nodes in the network). For unidimensional linear systems, the potential energy function can be obtained by taking the integral. However, when we have a multidimensional (non)linear system such as an Ising model or a GVAR model, taking the integral quickly becomes intractable. In such situations, we can approximate the generalized potential energy function (*U*) of each state of the system (*X*) based on the steady-state distribution ($P_{SS}$).

There are different ways to define the state of the system, *X*. We could be interested in the specific configuration of the values of the variables in the system, so-called *microstate* of the system. For instance, if we have a system with two binary variables, $x_1$ and $x_2$, than there are four possible microstates: $(x_1 = 0,\,x_2 = 0)$, $(x_1 = 1,\,x_2 = 0)$, $(x_1 = 0,\,x_2 = 1)$, $(x_1 = 1,\,x_2 = 1)$. However, in psychopathology, we are often interested in the *macrostate* of the system, e.g., the number of active symptoms, as it represents general symptom severity. For a system with two binary variables, there are three possible macrostates: the system’s sum score could be 0, 1, or 2. To define a generalized potential function for the macrostate of the system, we can sum over the potential energy function for all microstates with a similar number of active symptoms (Cui et al., 2023c), following:

4 $$\begin{aligned} \begin{aligned} U(\pmb {X})&= -\ln P_{SS}(\pmb {X}) \\&= -\ln \sum _{(\sum {x_i}) = \pmb {X}} P(x_i) \end{aligned} \end{aligned}$$

where $\pmb {X}$ are the possible *microstate* of the network. To get to the approximation of the generalized potential energy function, we take the negative logarithm of the steady-state distribution (Cui et al., 2023a, b, c; Wang et al., 2008). As we take the negative logarithm, states with lower density in the steady-state distribution have higher potential energy.

For the Ising model, we know the potential energy function. This function is typically denoted as the Hamiltonian, represented as *H*(*x*):

5 $$\begin{aligned} H(x) = - \sum _{\langle i,j \rangle } w_{i,j} x_i x_j - \sum _{i} \tau x_i \end{aligned}$$

The steady-state distribution needed to construct our stability landscape *P*(*x*), is then proportional to the exponent of *H*(*x*):

6 $$\begin{aligned} P(x) \propto e^{- H(x)} \end{aligned}$$

Similarly to the Ising model, to construct the stability landscape, we need to derive the steady state distribution of the GVAR model. The GVAR model assumes all parameters (e.g., the mean, variance, and auto-regressive parameters) are stationary across the measured time-period (Burger et al., 2022). The only exogenous random variable in the GVAR model is $\pmb {\varepsilon }$, which is Gaussian. As the model assumes stationarity, it follows that the response variable *y* is also Gaussian (Epskamp, 2020). This is a convenient property of the GVAR model, as this allows us to derive that the steady state distribution of a variable in the GVAR model is a Gaussian distribution with a mean of $\mu$ and variance of:

7 $$\begin{aligned} \textrm{vec} \left( \textrm{var}(\pmb {y})\right) = (\pmb {I} - \pmb {B} \otimes \pmb {B})^{-1} \textrm{vec}\left( \pmb {\Sigma }\right) \end{aligned}$$

where $\pmb {I}$ is an identity matrix containing ones on the diagonal and zeros on the off-diagonal elements.

In contrast to the Ising model where the macrostate is the number of active symptoms, we work with continuous variables and thus the macrostate refers to the sum score of the variables in the network. Thus, to obtain the potential landscape of the macrostate for the GVAR model, we take a normal distribution with a mean of the sum over the means of each variable in the system and a standard deviation of the square root of the sum over all implied variances.

Let $\textbf{y}_t$ represent an $n_v$ length vector with responses. We can model these responses using a lag-1 graphical vector autoregressive (GVAR) model:

$$ \textbf{y}_t = \pmb {\mu } + \textbf{B} (\textbf{y}_{t-1} - \pmb {\mu }) + \pmb {\zeta }_t. $$

Here, the *transpose* of $\textbf{B}$ is often used to draw a temporal network, and $\pmb {\mu }$ represents the mean or expected value of $\textbf{y}$. We assume that $\pmb {\zeta }_t$ is normally distributed:

$$ \pmb {\zeta }_T \sim \mathcal {N}(\textbf{0}, \pmb {\Sigma }). $$

Where the matrix $\pmb {\Sigma }$ represents the variance–covariance matrix of $\pmb {\zeta }$. This matrix can further be modeled as a Gaussian graphical model (GGM Epskamp et al., 2018b):

$$ \pmb {\Sigma } = \pmb {\Delta } (\textbf{I} - \pmb {\Omega })^{-1} \pmb {\Delta }, $$

in which $\pmb {\Omega }$ represents the partial correlation matrix with zeroes on the diagonal, which is typically used to draw a *contemporaneous* network. The $\pmb {\Delta }$ matrix is a diagonal scaling matrix that controls the variance.

Of note, $\pmb {\zeta }$ represents the *innovation* in the model and not, as commonly stated, a residual. A residual would indicate measurement error or something orthogonal to the process of interest. The innovation term, however, is only orthogonal to previous time points. Let *k* be an integer, then it follows that:

$$ \textrm{cov}(\pmb {\zeta }_T, \textbf{y}_{T + k}) = {\left\{ \begin{array}{ll} = \textbf{O} & \text {if } k < 0 \\ \ne \textbf{O} & \text {if } k \ge 0 \end{array}\right. } $$

Because $\pmb {\zeta }$ is the only exogenous random variable in this model, the model is linear, and $\pmb {\zeta }$ is Gaussian, it follows that $\textbf{y}$ is itself also Gaussian. We will assume the VAR model is *stationary*; which means that we assume the expected values and variance–covariance structure of $\textbf{y}$ is the same for any time point *t*:

$$ \begin{aligned} \mathbb {E}(\textbf{y}_T)&= \mathbb {E}(\textbf{y}_{T+k}) = \pmb {\mu } \quad \forall _k \\ \textrm{var}(\textbf{y}_T)&= \textrm{var}(\textbf{y}_{T+k}) = \pmb {\Sigma } \quad \forall _k, \end{aligned} $$

in which $\mathbb {E}(\ldots )$ represents the expected value and $\textrm{var}(\ldots )$ the variance–covariance matrix. We can then see that:

$$ \begin{aligned} \mathbb {E}(\textbf{y}_T)&= \mathbb {E}\left[ \pmb {\mu } + \textbf{B} (\textbf{y}_{t-1} - \pmb {\mu }) + \pmb {\zeta }_t \right] \\&= \pmb {\mu } + \textbf{B} \mathbb {E}(\textbf{y}_{t-1}) - \textbf{B} \mathbb {E}(\pmb {\mu }) + \mathbb {E}(\pmb {\zeta }_t) \\&= \pmb {\mu } + \textbf{B} \mathbb {E}(\textbf{y}_{t-1}) - \textbf{B} \mathbb {E}(\pmb {\mu }) + \textbf{0} \\&= \pmb {\mu } + \textbf{B} \pmb {\mu } - \textbf{B} \pmb {\mu } + \textbf{0} \\&= \pmb {\mu }. \end{aligned} $$

$$ \begin{aligned} \textrm{vec}(\pmb {\Sigma } - \textbf{B} \pmb {\Sigma } \textbf{B}^{\top })&= \textrm{vec}(\pmb {\Sigma }^{(\zeta )}) \\ \textrm{vec}(\pmb {\Sigma }) - (\textbf{B} \otimes \textbf{B}) \textrm{vec}(\pmb {\Sigma })&= \textrm{vec}(\pmb {\Sigma }^{(\zeta )}) \\ \left( \textbf{I} - \textbf{B} \otimes \textbf{B} \right) \textrm{vec}(\pmb {\Sigma })&= \textrm{vec}(\pmb {\Sigma }^{(\zeta )}) \\ \textrm{vec}(\pmb {\Sigma })&= \left( \textbf{I} - \textbf{B} \otimes \textbf{B} \right) ^{-1} \textrm{vec}(\pmb {\Sigma }^{(\zeta )}) \\ \pmb {\Sigma }&= \textrm{mat}\left[ \left( \textbf{I} - \textbf{B} \otimes \textbf{B} \right) ^{-1} \textrm{vec}(\pmb {\Sigma }^{(\zeta )})\right] . \end{aligned} $$

In this derivation, $\textrm{vec}$ represents the vectorization operator (stacking the columns), $\textrm{mat}$ its inverse that takes columns and makes a matrix (basically the matrix function in R), and $\otimes$ represents the Kronecker product.

With these expressions at hand, we can readily form the stationary density function of $\textbf{y}$ and use the negative log density to draw a stability landscape based on the item score.

We can use this to obtain the stability landscape for the sum score of the variables in the GVAR model. Let $s_y$ be the sum score at time point *t*:

$$ s_t = \textbf{1}^{\top } \textbf{y}_t = \sum _{v=1}^{n_v} y_{tv}, $$

in which $\textbf{1}$ is a vector of ones. We can then obtain the mean and variance of the sum score:

$$ \begin{aligned} \mu ^{(s)}&= \textbf{1}^{\top } \pmb {\mu } = \sum _{v=1}^{n_v} \mu _v \\ \pmb {\Sigma }^{(s)}&= \textbf{1}^{\top } \pmb {\Sigma } \textbf{1} = \sum _{i=1}^{n_v} \sum _{j=1}^{n_v} \sigma _{ij} \end{aligned} $$

## Footnotes

1 In the network theory of psychopathology, the thresholds are equivalent to the difficulty parameters in a Rasch model (Marsman et al., 2018).

2 Mathematically, these dynamics can be described using a cusp catastrophe (Finnemann et al., 2021a).

3 We ignore the $\beta$ parameter, so-called inverse temperature in physic literature, by setting it to one. We do so as the $\beta$ parameter is unidentifiable (that is, it is unobtainable when estimating the Ising model based on data).

4 While the edge weights have different interpretations, it is possible to transform the parameters of the Ising model in the $\{0,1\}$ domain to the $\{-1, 1\}$ domain and vice versa, making them statistically equivalent, as shown by Haslbeck et al. (2021b). However, changes in the parameters affect the dynamics of the model domains differently.

5 The covariance matrix, $\pmb {\Sigma }$, can be modeled as a Gaussian graphical model: $\pmb {\Sigma } = \pmb {\Delta } (\textbf{I} - \pmb {\Omega })^{-1} \pmb {\Delta }$, where $\pmb {\Omega }$ denotes the contemporaneous network, and $\pmb {\Delta }$ is a diagonal scaling matrix that controls the variance of the items.

6 Note that for these models, one could also derive the impact of changing parameters on means/variances. For the GVAR model, see the Appendix A for such a derivation.

7 Although the baseline network is derived from cross-sectional data, whereas the predictions of the network theory are based on idiographic networks, the parameterization is in line with the network theory as presented in Cramer et al. (2016). In the baseline Ising network within the $\{0,1\}$ domain, we have thresholds between -6 and -2 (which is comparable to the simulations in Cramer et al., 2016). Furthermore, according to the network theory, relations between symptoms are predominantly positive, as symptoms are more likely to exacerbate rather than alleviate each other (Borsboom et al., 2017).

8 Although mood disturbances can be part of a symptom (e.g., depressed mood in MDD), symptoms in diagnostic systems are typically defined by their persistence over a longer period (e.g., at least two weeks). In contrast, mood states measured in ESM studies fluctuate across hours or days. We therefore see no immediate logical reason to assume that a person with stronger causal connections between symptoms should also show higher (partial) correlations between momentary mood states.

## Code availability

The R code is publicly accessible through the following URL: https://osf.io/rgzqv/.

## References

[CR1] Aarts, E., & Haslbeck, J. (2025). Modeling psychological time series with multilevel hidden Markov models: A tutorial. 10.1037/met0000830 42113112

[CR2] Borsboom, D. (2017). A network theory of mental disorders. *World Psychiatry,* *16*(1), 5–13. 10.1002/wps.20375 PMC5269502 28127906

[CR3] Borsboom, D. et al. (2017). Mental disorders, network models, and dynamical systems. *Philosophical Issues in Psychiatry IV: Psychiatric Nosology DSM-5 (International Perspectives in Philosophy and Psychiatry)*, pp. 80–97.

[CR4] Borsboom, D., & Cramer, A. O. (2013). Network analysis: An integrative approach to the structure of psychopathology. *Annual Review of Clinical Psychology,**9*, 91–121. 23537483 10.1146/annurev-clinpsy-050212-185608

[CR5] Borsboom, D., Cramer, A. O., Fried, E. I., Isvoranu, A.-M., Robinaugh, D. J., Dalege, J., & van der Maas, H. L. (2022). Network perspectives. In *Network psychometrics with R: A Guide for Behavioral and Social Scientists,* (pp. 9–27). Routledge.

[CR6] Bringmann, L. F. (2021). Person-specific networks in psychopathology: Past, present, and future. *Current Opinion in Psychology,**41*, 59–64. 33862345 10.1016/j.copsyc.2021.03.004

[CR7] Bringmann, L. F., Helmich, M., Eronen, M., & Voelkle, M. (2023). Complex systems approaches to psychopathology. In R. F. Krueger & P. H. Blaney (Eds.), *Oxford Textbook of Psychopathology* (4th ed., pp. 103–122). Oxford: Oxford University Press.

[CR8] Bringmann, L. F., Ariens, S., Ernst, A. F., Snippe, E., & Ceulemans, E. (2024). Changing networks: Moderated idiographic psychological networks. *Advances in Psychology,* *2*, Article e658296.

[CR9] Burger, J., Hoekstra, R. H. A., Mansueto, A. C., & Epskamp, S. (2022). Network estimation from time series and panel data. In A.-M. Isvoranu, S. Epskamp, L. J. Waldorp, & D. Borsboom (Eds.), *Network psychometrics with R: A Guide for Behavioral and Social Scientists* (pp. 169–192). Routledge.

[CR10] Cramer, A. O., Van Borkulo, C. D., Giltay, E. J., Van Der Maas, H. L., Kendler, K. S., Scheffer, M., & Borsboom, D. (2016). Major depression as a complex dynamic system. *PloS One,**11*(12), Article e0167490. 27930698 10.1371/journal.pone.0167490 PMC5145163

[CR11] Cui, J., Hasselman, F., & Lichtwarck-Aschoff, A. (2023a). *Unlocking nonlinear dynamics and multistability from intensive longitudinal data: A novel method*. Psychological Methods: Advance online publication. 10.1037/met0000623 38095993

[CR12] Cui, J., Lichtwarck-Aschoff, A., Olthof, M., Li, T., & Hasselman, F. (2023b). From metaphor to computation: Constructing the potential landscape for multivariate psychological formal models. *Multivariate Behavioral Research,* *58*(4), 743–761. 10.1080/00273171.2022.2119927 36223116

[CR13] Cui, J., Lunansky, G., Lichtwarck-Aschoff, A., Mendoza, N., & Hasselman, F. (2023c). Quantifying the stability landscapes of psychological networks. PsyArXiv. 10.3758/s13428-025-02917-7 PMC12923465 41721170

[CR14] Cui, J., Wagenmakers, D., van Doorn, G. S., Hasselman, F., & Lichtwarck-Aschoff, A. (2025). Analyzing formal dynamic models in psychology: A tutorial using graphical tools. 10.3758/s13428-026-03077-y PMC13287130 42332294

[CR15] Dalege, J., Borsboom, D., Van Harreveld, F., Van den Berg, H., Conner, M., & Van der Maas, H. L. (2016). Toward a formalized account of attitudes: The causal attitude network (CAN) model. *Psychological Review,**123*(1), 2–22. 26479706 10.1037/a0039802

[CR16] De Vos, S., Wardenaar, K. J., Bos, E. H., Wit, E. C., Bouwmans, M. E., & De Jonge, P. (2017). An investigation of emotion dynamics in major depressive disorder patients and healthy persons using sparse longitudinal networks. *PLoS One,**12*(6), Article e0178586. 28570696 10.1371/journal.pone.0178586 PMC5453553

[CR17] Epskamp, S. (2020). Psychometric network models from time-series and panel data. *Psychometrika,**85*(1), 206–231. 32162233 10.1007/s11336-020-09697-3 PMC7186258

[CR18] Epskamp, S., & Fried, E. I. (2023). Mlvar: Multi-level vector autoregression. Retrieved from https://cran.r-project.org/package=bootnet

[CR19] Epskamp, S., van Borkulo, C. D., van der Veen, D. C., Servaas, M. N., Isvoranu, A.-M., Riese, H., & Cramer, A. O. (2018a). Personalized network modeling in psychopathology: The importance of contemporaneous and temporal connections. *Clinical Psychological Science,* *6*(3), 416–427. 10.1177/2167702617744325 PMC5952299 29805918

[CR20] Epskamp, S., Waldorp, L. J., Mõttus, R., & Borsboom, D. (2018b). The Gaussian graphical model in cross-sectional and time-series data. *Multivariate Behavioral Research,* *53*(4), 453–480. 10.1080/00273171.2018.1454823 29658809

[CR21] Finnemann, A., Borsboom, D., Epskamp, S., & van der Maas, H. L. J. (2021a). The theoretical and statistical Ising model: A practical guide in R. *Psych,* *3*(4), 594–618. 10.3390/psych3040039

[CR22] Finnemann, A., Borsboom, D., Epskamp, S., & van der Maas, H. L. (2021). The theoretical and statistical Ising model: A practical guide in R. *Psych,**3*(04), 593–617.

[CR23] Fried, E. I. (2020). Lack of theory building and testing impedes progress in the factor and network literature. *Psychological Inquiry,**31*(4), 271–288.

[CR24] Frumkin, M. R., Piccirillo, M. L., Beck, E. D., Grossman, J. T., & Rodebaugh, T. L. (2021). Feasibility and utility of idiographic models in the clinic: A pilot study. *Psychotherapy Research,**31*(4), 520–534. 10.1080/10503307.2020.1805133 PMC7902742 32838671

[CR25] Grynkiv, G., & Stentoft, L. (2018). Stationary threshold vector autoregressive models. *Journal of Risk and Financial Management,**11*(3), 45.

[CR26] Hamaker, E. L., & Grasman, R. P. (2015). To center or not to center? Investigating inertia with a multilevel autoregressive model. *Frontiers in Psychology,**5*, 1492. 25688215 10.3389/fpsyg.2014.01492 PMC4310502

[CR27] Hamaker, E. L., Grasman, R. P., & Kamphuis, J. H. (2016). Modeling bas dysregulation in bipolar disorder: Illustrating the potential of time series analysis. *Assessment,**23*(4), 436–446. 26906639 10.1177/1073191116632339

[CR28] Hamilton, J. D. (1994). *Time Series Analysis*. Princeton, NJ: Princeton University Press.

[CR29] Haslbeck, J. M. B., Bringmann, L. F., & Waldorp, L. J. (2021a). A tutorial on estimating time-varying vector autoregressive models. *Multivariate Behavioral Research,* *56*(1), 120–149. 10.1080/00273171.2020.1743630 32324066

[CR30] Haslbeck, J. M. B., Epskamp, S., Marsman, M., & Waldorp, L. J. (2021b). Interpreting the Ising model: The input matters. *Multivariate Behavioral Research,* *56*(2), 303–313. 10.1080/00273171.2020.1730150 32162537

[CR31] Haslbeck, J. M. B., Ryan, O., Robinaugh, D. J., Waldorp, L. J., & Borsboom, D. (2022). Modeling psychopathology: From data models to formal theories. *Psychological Methods,**27*(6), 930–957. 10.1037/met0000303 PMC10259162 34735175

[CR32] Haslbeck, J. M. B., Ryan, O., & Dablander, F. (2023). Multimodality and skewness in emotion time series. *Emotion,**8*, 2117–2141. 10.1037/emo0001218 37166827

[CR33] Heino, M. T., Proverbio, D., Marchand, G., Resnicow, K., & Hankonen, N. (2023). Attractor landscapes: A unifying conceptual model for understanding behaviour change across scales of observation. *Health Psychology Review,**17*(4), 655–672. 36420691 10.1080/17437199.2022.2146598 PMC10261543

[CR34] Henry, T. R., Robinaugh, D. J., & Fried, E. I. (2022). On the control of psychological networks. *Psychometrika,* *87*(1), 188–213. 10.1007/s11336-021-09796-9 PMC9205512 34390455

[CR35] Ising, E. (1925). Beitrag zur theorie des ferromagnetismus zeit. fur physik 31.

[CR36] Kalisch, R., Cramer, A. O., Binder, H., Fritz, J., Leertouwer, I., Lunansky, G., & Van Harmelen, A.-L. (2019). Deconstructing and reconstructing resilience: A dynamic network approach. *Perspectives on Psychological Science,**14*(5), 765–777. 31365841 10.1177/1745691619855637

[CR37] Kelley, S. W., Fisher, A. J., Lee, C. T., Gallagher, E., Hanlon, A. K., Robertson, I. H., & Gillan, C. M. (2023). Elevated emotion network connectivity is associated with fluctuations in depression. *Proceedings of the National Academy of Sciences,**120*(45), Article e2216499120. 10.1073/pnas.2216499120 PMC10636367 37903279

[CR38] Kendler, K. S., & Prescott, C. A. (1999). A population-based twin study of lifetime major depression in men and women. *Archives of General Psychiatry,**56*(1), 39–44. 9892254 10.1001/archpsyc.56.1.39

[CR39] Koval, P., & Kuppens, P. (2012). Changing emotion dynamics: Individual differences in the effect of anticipatory social stress on emotional inertia. *Emotion,**12*(2), 256–267. 21787072 10.1037/a0024756

[CR40] Kuppens, P., Allen, N. B., & Sheeber, L. B. (2010). Emotional inertia and psychological maladjustment. *Psychological Science,**21*(7), 984–991. 20501521 10.1177/0956797610372634 PMC2901421

[CR41] Koval, P., Kuppens, P., Allen, N. B., & Sheeber, L. (2012). Getting stuck in depression: The roles of rumination and emotional inertia. *Cognition & Emotion,**26*(8), 1412–1427. 22671768 10.1080/02699931.2012.667392

[CR42] Lunansky, G., Hoekstra, R. H., & Blanken, T. F. (2023). Disentangling the role of affect in the evolution of depressive complaints using complex dynamical networks. *Collabra: Psychology,**9*(1), 74841.

[CR43] Lydon-Staley, D. M., Xia, M., Mak, H. W., & Fosco, G. (2019). Adolescent emotion network dynamics in daily life and implications for depression. *Journal of Abnormal Child Psychology,**47*, 717–729. 10.1007/s10802-018-0474-y PMC6411453 30203118

[CR44] Marsman, M., Borsboom, D., Kruis, J., Epskamp, S., van Bork, R. V, Waldorp, L., & Maris, G. (2018). An introduction to network psychometrics: Relating Ising network models to item response theory models. *Multivariate Behavioral Research,* *53*(1), 15–35. 10.1080/00273171.2017.1379379 29111774

[CR45] McElroy, E., Napoleone, E., Wolpert, M., & Patalay, P. (2019). Structure and connectivity of depressive symptom networks corresponding to early treatment response. *EClinicalMedicine,**8*, 29–36. 10.1016/j.eclinm.2019.02.009 PMC6537518 31193604

[CR46] McGhie, S. F., & McNally, R. J. (2023). *Posttraumatic stress disorder symptoms and positive affect: Individual and multilevel dynamic networks*. Psychological Trauma: Theory, Research, Practice, and Policy. 10.1037/tra0001605 37843525

[CR47] Olthof, M., Hasselman, F., Oude Maatman, F., Bosman, A. M., & Lichtwarck-Aschoff, A. (2023). Complexity theory of psychopathology. *Journal of Psychopathology and Clinical Science,**132*(3), 314–323. 37126062 10.1037/abn0000740

[CR48] Pe, M. L., Kircanski, K., Thompson, R. J., Bringmann, L. F., Tuerlinckx, F., Mestdagh, M., Jonides, J., et al. (2015). Emotion-network density in major depressive disorder. *Clinical Psychological Science,**3*(2), 292–300. 31754552 10.1177/2167702614540645 PMC6871506

[CR49] Piccirillo, M. L., & Rodebaugh, T. L. (2022). Personalized networks of social anxiety disorder and depression and implications for treatment. *Journal of Affective Disorders,**298*, 262–276. 34699851 10.1016/j.jad.2021.10.034 PMC8690310

[CR50] Rabiner, L. R. (1989). A tutorial on hidden Markov models and selected applications in speech recognition. *Proceedings of the IEEE,**77*(2), 257–286.

[CR51] Robinaugh, D. J., Hoekstra, R. H., Toner, E. R., & Borsboom, D. (2020). The network approach to psychopathology: A review of the literature 2008–2018 and an agenda for future research. *Psychological Medicine,**50*(3), 353–366. 31875792 10.1017/S0033291719003404 PMC7334828

[CR52] Ryan, O., Dablander, F., & Haslbeck, J. (2023). Towards a generative model for emotion dynamics. PsyArXiv. 10.31234/osf.io/x52ns 10.1037/rev0000513 40146601

[CR53] Schweren, L., Van Borkulo, C. D., Fried, E., & Goodyer, I. M. (2018). Assessment of symptom network density as a prognostic marker of treatment response in adolescent depression. *JAMA Psychiatry,**75*(1), 98–100. 10.1001/jamapsychiatry.2017.3561 PMC5833532 29188277

[CR54] Shin, K. E., Newman, M. G., & Jacobson, N. C. (2022). Emotion network density is a potential clinical marker for anxiety and depression: Comparison of ecological momentary assessment and daily diary. *British Journal of Clinical Psychology,**61*, 31–50. 33963538 10.1111/bjc.12295 PMC8572316

[CR55] Sturt, E. (1981). Hierarchical patterns in the distribution of psychiatric symptoms. *Psychological Medicine,**11*(4), 783–794. 7323234 10.1017/s0033291700041283

[CR56] Suls, J., Green, P., & Hillis, S. (1998). Emotional reactivity to everyday problems, affective inertia, and neuroticism. *Personality and Social Psychology Bulletin,**24*(2), 127–136.

[CR57] van Bork, R., Rhemtulla, M., Waldorp, L. J., Kruis, J., Rezvanifar, S., & Borsboom, D. (2021). Latent variable models and networks: Statistical equivalence and testability. *Multivariate Behavioral Research,**56*(2), 175–198. 31617420 10.1080/00273171.2019.1672515

[CR58] van Borkulo, C., Boschloo, L., Borsboom, D., Penninx, B. W., Waldorp, L. J., & Schoevers, R. A. (2015). Association of symptom network structure with the course of depression. *JAMA Psychiatry,**72*(12), 1219–1226. 26561400 10.1001/jamapsychiatry.2015.2079

[CR59] Van der Maas, H. L., Dalege, J., & Waldorp, L. (2020). The polarization within and across individuals: The hierarchical Ising opinion model. *Journal of Complex Networks,* *8*(2), cnaa010. 10.1093/comnet/cnaa010

[CR60] van der Maas, H. L., Borsboom, D., & Waldorp, L. (2025). *The statistical physics of psychological networks: Zero matters*. 10.31219/osf.io/7wfqj_v1 10.1037/rev0000611 41701267

[CR61] Van Dongen, N., van Bork, R., Finnemann, A., van der Maas, H., Robinaugh, D., Haslbeck, J. M. B., & Borsboom, D. (2024). *Productive explanation: A framework for evaluating explanations in psychological science*. Psychological Review: Advance online publication. 10.1037/rev0000479 39023936

[CR62] Wang, J., Xu, L., & Wang, E. (2008). Potential landscape and flux framework of nonequilibrium networks: Robustness, dissipation, and coherence of biochemical oscillations. *Proceedings of the National Academy of Sciences,**105*(34), 12271–12276. 10.1073/pnas.0800579105 PMC2527901 18719111

[CR63] Wichers, M., Schreuder, M. J., Goekoop, R., & Groen, R. N. (2019). Can we predict the direction of sudden shifts in symptoms? transdiagnostic implications from a complex systems perspective on psychopathology. *Psychological Medicine,**49*(3), 380–387. 30131079 10.1017/S0033291718002064 PMC6331686

[CR64] Wigman, J., Van Os, J., Borsboom, D., Wardenaar, K., Epskamp, S., Klippel, A., Wichers, M., et al. (2015). Exploring the underlying structure of mental disorders: Cross-diagnostic differences and similarities from a network perspective using both a top-down and a bottom-up approach. *Psychological Medicine,**45*(11), 2375–2387. 25804221 10.1017/S0033291715000331

[CR65] Zhang, Y., Revol, J., Lafit, G., Ernst, A., Razum, J., Ceulemans, E., & Bringmann, L. (2025). Meeting the bare minimum: Quality assessment of idiographic temporal networks using power analysis and predictive accuracy analysis. *Advances in Methods and Practices in Psychological Science,**8*(4), 25152459251372116.

